# A Rare Case of Cerebral Amyloid Angiopathy-Related Inflammation With Subarachnoid Diffusion Restriction Mimicking Rheumatoid Meningitis

**DOI:** 10.7759/cureus.97214

**Published:** 2025-11-19

**Authors:** Hiroyuki Maki, Yuka Kondo, Sachiko Minamiguchi, Daijiro Kojima, Shigeo Ohba, Shunsuke Adachi, Hirohisa Watanabe, Masanori Inoue

**Affiliations:** 1 Department of Radiology, Fujita Health University School of Medicine, Toyoake, JPN; 2 Department of Diagnostic Pathology, Fujita Health University School of Medicine, Toyoake, JPN; 3 Department of Neurosurgery, Fujita Health University School of Medicine, Toyoake, JPN; 4 Department of Neurology, Fujita Health University School of Medicine, Toyoake, JPN

**Keywords:** amyloid beta-related angiitis, caa-related inflammation, cerebral amyloid angiopathy, diffusion-weighted imaging, primary angiitis cns, rheumatoid meningitis

## Abstract

Some patients with cerebral amyloid angiopathy (CAA), which is characterized by amyloid β fibril deposition in cortical and leptomeningeal vessels, develop an inflammatory response, leading to CAA-related inflammation (CAA-RI). In such cases, histopathologically confirmed vasculitis is defined as amyloid β-related angiitis (ABRA). Here, we report the rare case of an 80-year-old woman who presented with anomic aphasia and mild right upper limb weakness. Magnetic resonance imaging revealed restricted diffusion in the subarachnoid space along the cerebral sulci and dura mater, mainly within the left parietal lobe. Additional imaging findings included lobar microbleeds, edematous white matter changes, and leptomeningeal and dural enhancement. Brain biopsy revealed vasculitis with amyloid β deposition, consistent with ABRA. Marked lymphoplasmacytic infiltration, which was present in the leptomeninges and the dural surface, was considered the cause of the subarachnoid diffusion restriction. Immunosuppressive treatment led to clinical and radiological improvement. As illustrated in this case, CAA-RI may present with atypical findings of subarachnoid diffusion restriction and dural involvement, and the recognition of these features, which can mimic rheumatoid meningitis and other inflammatory conditions, is important for differential diagnosis.

## Introduction

Cerebral amyloid angiopathy (CAA) is an age-related small-vessel disease pathologically characterized by the progressive deposition of amyloid-β (Aβ) fibrils within the walls of small- and medium-sized arteries and capillaries in cortical and leptomeningeal vessels [1]. Among Aβ isoforms, Aβ40 is predominantly associated with vascular deposition in CAA, whereas Aβ42 accumulates mainly in neuritic plaques of patients with Alzheimer’s disease (AD) [2]. The prevalence of CAA markedly increases with age, reaching nearly 80% in individuals over 80 years of age [3]. The prevalence and severity of CAA pathology tend to be worse among patients with AD [4]. A major mechanism for the clearance of cerebral Aβ is the intramural peri-arterial drainage (IPAD) pathway, in which interstitial fluid is propelled along basement membranes within the tunica media by arterial pulsations, moving in the reverse direction to blood flow and eventually draining into the cerebrospinal fluid (CSF) and cervical lymph nodes [1]. The distribution of vascular Aβ deposition closely corresponds to the IPAD route. Arterial pulsatility and vascular reactivity decline with aging, arteriosclerosis, and progressive vascular Aβ accumulation, resulting in impaired IPAD-mediated Aβ clearance and progression of CAA [5]. The representative clinical manifestations and imaging findings of CAA are incorporated into the Boston criteria version 2.0, published in 2022 [6]. A diagnosis of probable CAA can be made in individuals over 50 years of age who present with spontaneous intracerebral hemorrhage (ICH), transient focal neurological episodes, or cognitive impairment and who exhibit two or more strictly lobar hemorrhagic lesions (i.e., lobar cerebral microbleeds, ICH, or cortical superficial siderosis/subarachnoid hemorrhage) on blood-sensitive magnetic resonance imaging (MRI). The Boston criteria version 2.0 further includes non-hemorrhagic MRI signs, namely, severe perivascular spaces in the centrum semiovale and the subcortical multi-spot pattern of white matter hyperintensities, in the diagnosis of CAA. The inclusion of non-hemorrhagic signs in patients with a single lobar hemorrhagic lesion enhances the diagnostic sensitivity for probable CAA while maintaining high specificity [6].

Some patients exhibit an inflammatory response at sites of Aβ deposition, a condition termed CAA-related inflammation (CAA-RI), which is histopathologically characterized by lymphoplasmacytic infiltration around amyloid-laden vessels. This feature may extend into the vessel wall in severe cases, resulting in structural destruction consistent with vasculitis, termed Aβ-related angiitis (ABRA) [7]. CAA-RI shares pathophysiologic similarities with amyloid-related imaging abnormalities that occur during treatment with anti-amyloid monoclonal antibody in patients with AD [8]. Clinically, CAA-RI presents with an acute or subacute onset of headache, decreased level of consciousness, behavioral changes, focal neurologic deficits, and seizures not associated with ICH [9]. The diagnostic criteria for probable CAA-RI are as follows: age >40 years; acute or subacute onset of symptoms; asymmetric subcortical white matter hyperintensities on MRI with or without leptomeningeal enhancement; coexistence of hemorrhagic lesions fulfilling the Boston criteria for CAA; and exclusion of neoplastic, infectious, and systemic autoimmune diseases [10]. The principal differential diagnoses of CAA-RI are meningitis and primary angiitis of the central nervous system (PACNS) [9]. High-dose corticosteroids remain the mainstay of initial therapy for CAA-RI [11]. In steroid-refractory cases, additional immunosuppressive agents, including cyclophosphamide and azathioprine, may be considered to achieve disease control [12]. When appropriately managed, most patients with CAA-RI experience a favorable clinical course. However, disease recurrence remains a major concern. Recurrence was observed in 26% of patients who received immunosuppressive therapy, which was lower than the rate of 71% observed among untreated individuals, emphasizing the importance of early recognition and appropriate immunomodulatory management [13]. Here, we present a rare case of CAA-RI with subarachnoid diffusion restriction and discuss its underlying pathophysiology.

## Case presentation

An 80-year-old woman was referred to our hospital with a one-month history of anomic aphasia as the chief complaint. Neurologic examination revealed altered speech and mild weakness in the right upper limb, with a manual muscle test grade of 4. She had no history of cognitive impairment or rheumatoid arthritis. CSF analysis revealed a mildly elevated protein level (94 mg/dL), normal glucose level, cell counts within normal limits, and no red blood cells. Infectious etiologies, including viral and bacterial meningitides, were excluded. Both serum and CSF were negative for anti-cyclic citrullinated peptide (CCP) antibodies. Serum was also negative for rheumatoid factor and anti-neutrophil cytoplasmic antibodies, and serum IgG4 level was within the normal range. Cranial computed tomography revealed low attenuation in the white matter of the left parietal and occipital lobes.

**Figure 1:**
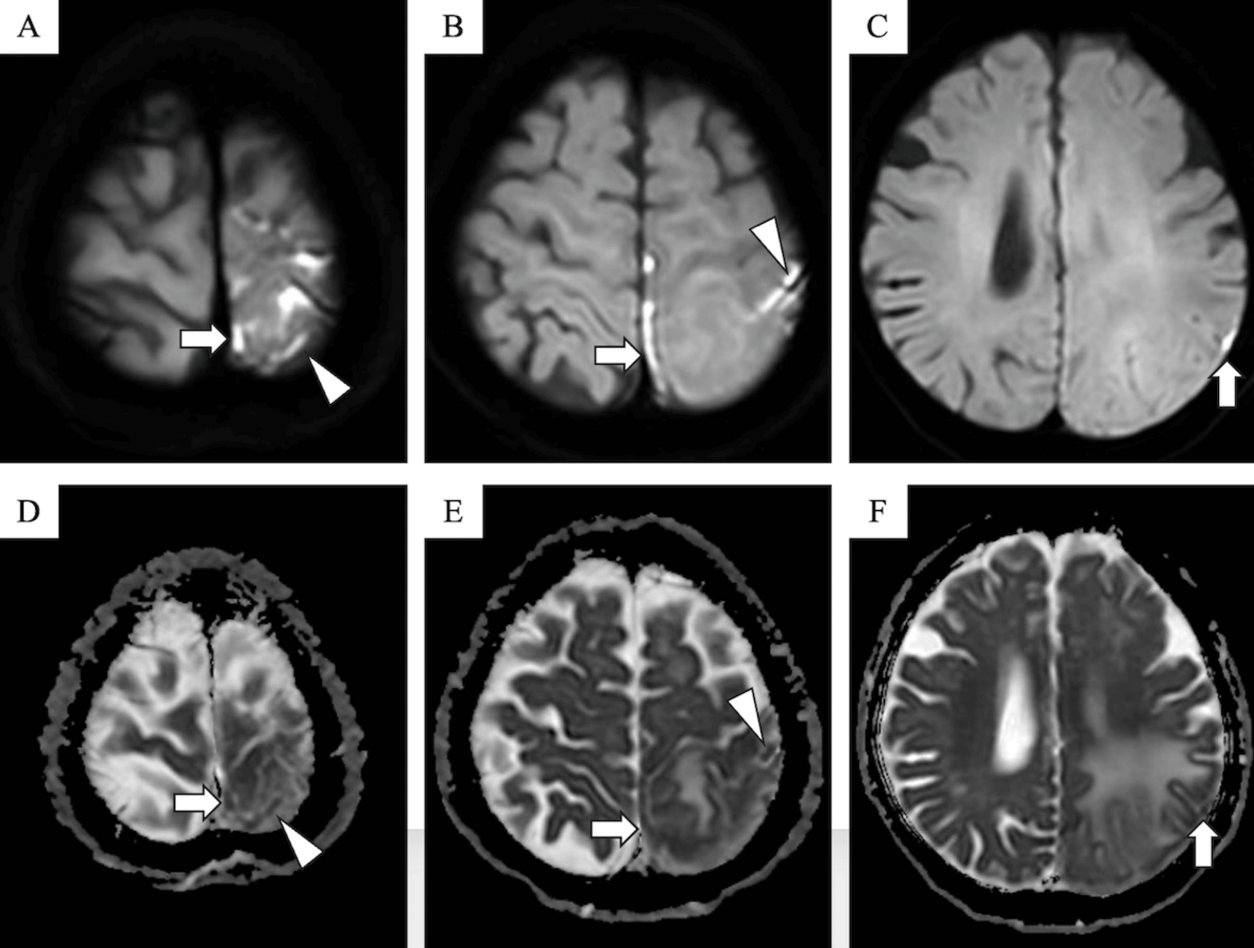
Diffusion-weighted imaging and apparent diffusion coefficient mapping. (A–C) Diffusion-weighted imaging shows hyperintensities within the subarachnoid space along the cerebral sulci (arrowheads) and the dura mater (arrow). (D–F) Apparent diffusion coefficient maps show corresponding reduced values.

The patient was evaluated with cranial MRI. By diffusion-weighted imaging (DWI), multiple focal hyperintensity was observed along the cerebral sulci of the left parietal and occipital lobes and the dura mater, accompanied by a corresponding reduction in apparent diffusion coefficients (Figure 1).

**Figure 2:**
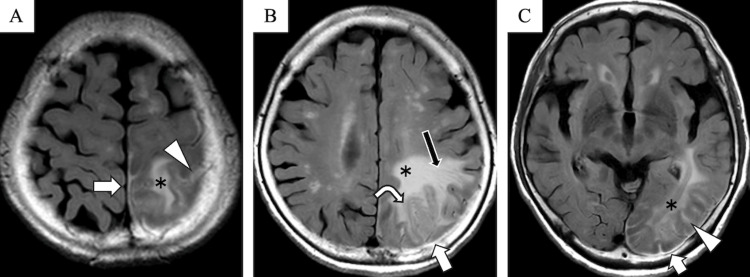
Fluid-attenuated inversion recovery imaging. Fluid-attenuated inversion recovery imaging shows hyperintensities within the subarachnoid space along the sulci of the parietal and occipital lobes (A–C, arrowhead) and the adjacent dura mater (A–C, arrow), extending beyond the areas of hyperintensity on diffusion-weighted imaging. Additionally, an area of hyperintensity is seen extending from the subcortical white matter to deep white matter of the parietal and occipital lobes (A–C, asterisk), accompanied by dilated perivascular spaces (B, black arrow) and swelling of the left superior  parietal cortex (B, curved arrow).

T2-weighted imaging (T2WI) and fluid-attenuated inversion recovery (FLAIR) showed hyperintense signals involving the left parietal and occipital sulci and the adjacent dura mater, extending beyond the region of diffusion restriction (Figure 2). In addition, T2WI and FLAIR showed diffuse hyperintensity extending from the subcortical white matter to the deep white matter of the left parietal and occipital lobes, accompanied by dilated perivascular spaces (Figure 2). Mild swelling and hyperintensity were observed in the cortex of the left superior parietal lobule (Figure 2).

**Figure 3:**
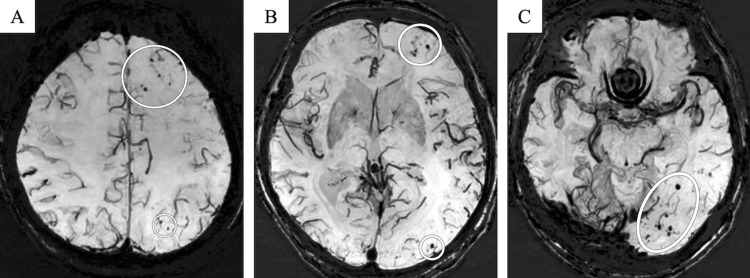
Susceptibility-weighted imaging. Minimum intensity projection images obtained with susceptibility-weighted imaging show lobar microbleeds in the left parietal, occipital, and frontal lobes (A–C, circles). No subarachnoid hemorrhage is observed on susceptibility-weighted imaging.

Susceptibility-weighted imaging (SWI) revealed lobar microbleeds not only in the left parietal and occipital lobes, corresponding to the diffuse hyperintensity observed with T2WI and FLAIR, but also in the frontal lobes, albeit without associated white matter lesions (Figure 3). Subarachnoid hemorrhage was not detected on SWI.

**Figure 4:**
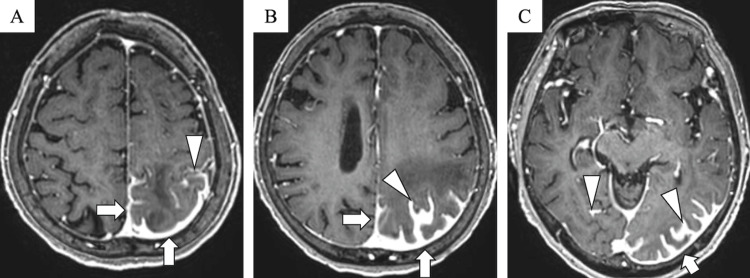
Contrast-enhanced T1-weighted imaging. Contrast-enhanced T1-weighted imaging shows leptomeningeal enhancement extending from the parietal to the occipital lobe (A–C, arrowhead) and enhancement of the adjacent dura mater (A–C, arrow). No parenchymal enhancement is observed.

Contrast-enhanced T1-weighted imaging (T1WI) revealed leptomeningeal enhancement extending from the left parietal to the occipital lobes, as well as enhancement of the adjacent dura mater (Figure 4). Focal leptomeningeal enhancement was also seen in the right occipital lobe (Figure 4). No contrast enhancement was observed in the brain parenchyma.

Further imaging did not reveal abnormalities in lacrimal and salivary glands, nasal cavity, paranasal sinuses, or thoracic and abdominal organs. Based on these imaging findings, the differential diagnoses included CAA-RI, rheumatoid meningitis, PACNS, anti-neutrophil cytoplasmic antibody-associated vasculitis, and IgG4-related disease. The presence of lobar microbleeds on SWI and the laboratory test results suggested CAA-RI; however, subarachnoid diffusion restriction was considered atypical for CAA-RI. Therefore, a brain biopsy was performed.

**Figure 5:**
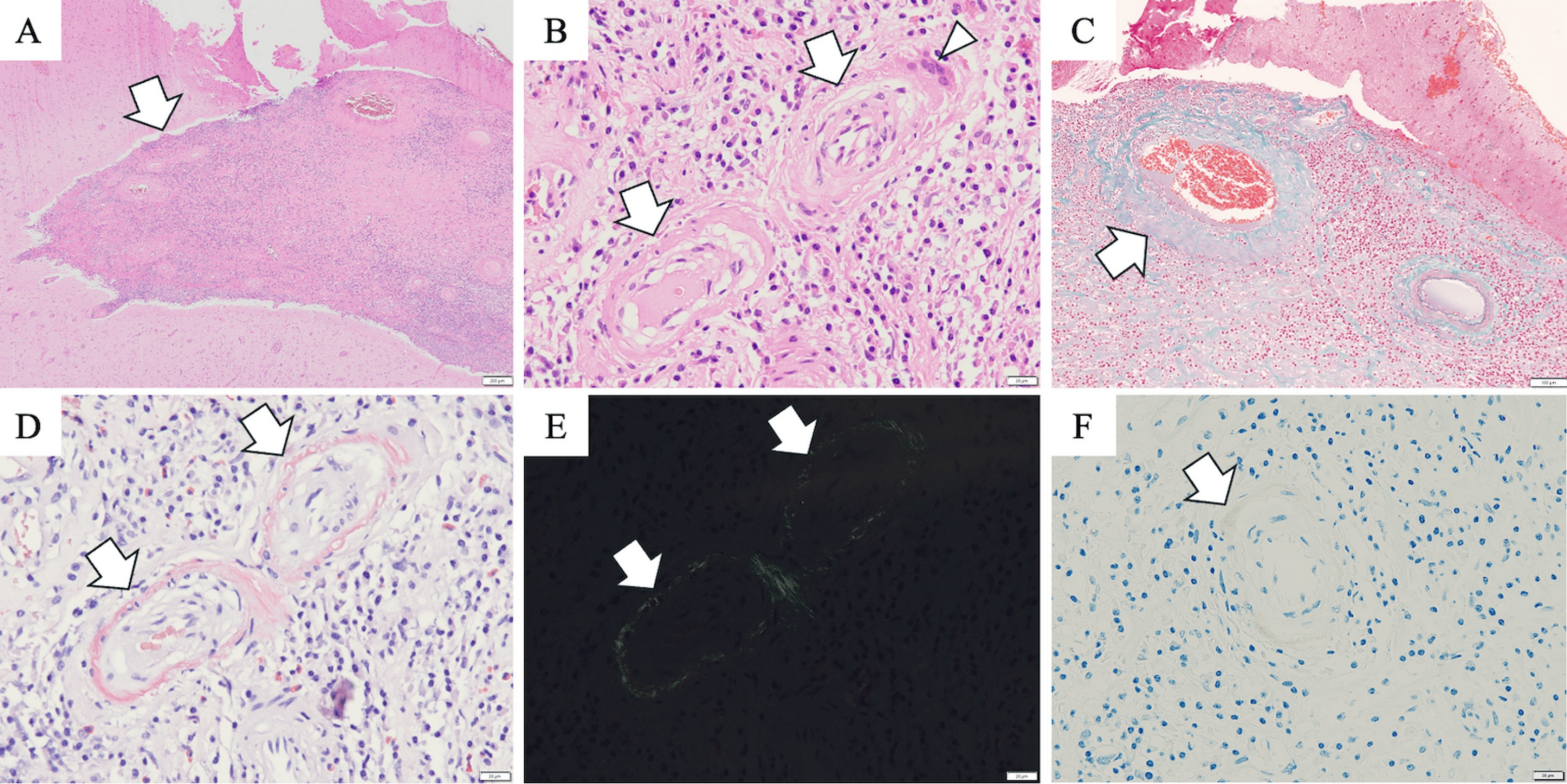
Histopathologic assessment of the brain biopsy specimen. (A, B) Hematoxylin/eosin staining shows dense lymphoplasmacytic infiltration within the leptomeninges (A, arrow). Leptomeningeal vessels exhibit a double-barrel appearance (B, arrows) with multinucleated giant cells (B, arrowhead). (C) Elastica–Masson staining shows inflammatory disruption of the vascular wall and fibrinoid necrosis (C, arrow). (D, E) Congo red staining shows amyloid β deposits within the vessel walls (D, arrows). Under polarized light, these deposits exhibit apple-green birefringence, confirming the presence of amyloid β deposits in the vessel walls (E, arrows). (F) Amyloid-β deposits are confirmed by immunostaining (F, arrow). Scale bars: 200 μm (A), 20 μm (B, D–F), 100 μm (C)

Specimens were collected from the left superior parietal lobule, which included cortical lesions, as well as the adjacent dura mater. Histopathologic examination revealed dense lymphoplasmacytic infiltration within the leptomeninges, accompanied by the transmural inflammation of leptomeningeal and parenchymal vessels (Figure 5). Elastica-Masson staining showed inflammatory destruction of the vascular wall structure (Figure 5). Congo red staining and immunohistochemistry confirmed Aβ deposition within the vascular walls (Figure 5). No infarcts were identified in the brain parenchyma.

**Figure 6:**
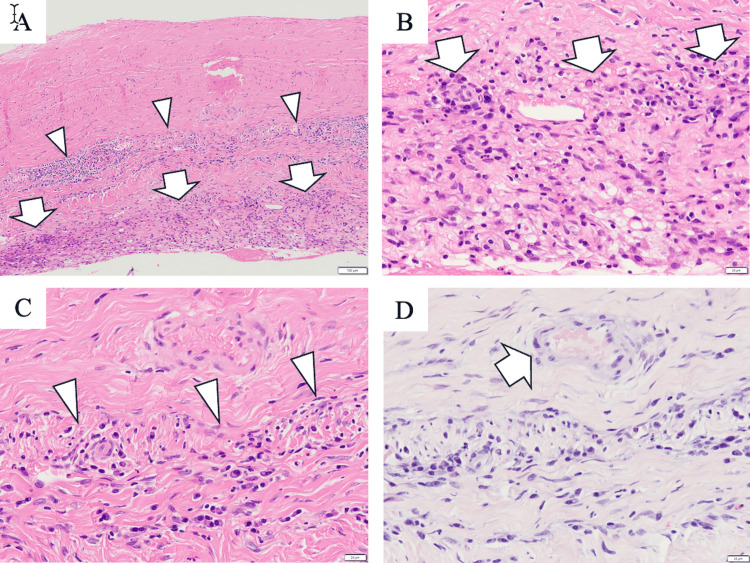
Histopathologic assessment of the dural biopsy specimen. A–C) Hematoxylin/eosin staining shows dense lymphoplasmacytic infiltration along the dural surface (A and B, arrows) and within the dura mater (A and C, arrowheads). (D) Congo red staining shows no amyloid deposition in vessel walls within the dura mater (arrow). Scale bars: 100 μm (A), 20 μm (B–D)

Marked lymphoplasmacytic infiltration was also observed on the dural surface (Figure 6). Histopathologic examination of the dura mater revealed lymphoplasmacytic infiltration; however, there was no evidence of vasculitis or Aβ deposition (Figure 6).

The IgG4/IgG-positive cell ratio was 22.5%, ruling out the diagnosis of IgG4-related disease. Based on these findings, the patient was diagnosed with CAA-RI, with the leptomeningeal and parenchymal lesions indicating ABRA.

Steroid pulse therapy was initiated with intravenous methylprednisolone, administered at a dose of 1,000 mg/day for three days, followed by maintenance therapy with methylprednisolone at a dose of 35 mg/day. Following the transient worsening of white matter edema, which was observed on serial biweekly MRI scans obtained after the biopsy, tacrolimus was introduced at a dose of 1 mg/day. Subsequently, the white matter edema gradually resolved, accompanied by the clinical improvement of language function and muscle weakness. The patient was discharged on day 48 after treatment initiation, with no recurrence of symptoms thereafter.

## Discussion

CAA typically presents after 50 years of age, with clinical symptoms such as intracerebral hemorrhage, transient focal neurological episodes, and cognitive impairment. The second version of the Boston criteria is widely used for the diagnosis of CAA [6]. In these patients, Aβ fibrils deposited in vessel walls may occasionally provoke an inflammatory response, resulting in the onset of CAA-RI. Typical clinical manifestations of CAA-RI include cognitive impairment, focal neurological signs, headache, and seizures. Neuroimaging findings of lobar microbleeds in combination with edematous white matter lesions and leptomeningeal enhancement are highly suggestive signs of CAA-RI [14]. Although the definitive diagnosis of CAA-RI requires histopathologic confirmation, the validated 2016 diagnostic criteria allow “probable” or “possible” as diagnostic classes based on clinical and MRI findings [10]. The patient presented here fulfilled the criteria for probable CAA-RI; however, the presence of diffusion restriction within the subarachnoid space, accompanied by marked dural enhancement, was atypical, prompting brain biopsy to exclude other diagnoses. The histopathologic examination of the biopsy specimen led to the identification of ABRA in the leptomeningeal and parenchymal vessels, establishing the definitive diagnosis of CAA-RI. Pronounced lymphoplasmacytic infiltration was observed within the leptomeninges and along the dural surface, corresponding to the diffusion restriction observed on MRI. Although infiltration was also found within the dura mater, Aβ deposition or vasculitis was not observed in the intradural vessels. Overall, these findings suggested that the dural and peridural inflammatory changes were secondary to leptomeningeal CAA-RI. While dural enhancement has been reported in patients with CAA-RI [15], to our knowledge, this is the first case demonstrating diffusion restriction within the subarachnoid space.

Rheumatoid meningitis, a rare central nervous system complication of rheumatoid arthritis characterized by chronic inflammation of the meninges [16], is an important condition that should be included in the differential diagnosis of the present case. Patients with rheumatoid meningitis are positive for either serum rheumatoid factor or serum anti-CCP antibodies [17]. Histopathologically, rheumatoid meningitis is characterized by lymphoplasmacytic inflammation with multinucleated giant cells in the meninges and subarachnoid space, rheumatoid nodules, and small-vessel vasculitis; however, inflammatory changes are minimal in the underlying brain parenchyma [16]. On MRI, asymmetric or unilateral subarachnoid diffusion restriction, reflecting marked inflammatory cell infiltration, is considered a hallmark finding of rheumatoid meningitis [18]. Additional findings include FLAIR hyperintensity in the subarachnoid space in addition to thickening and contrast enhancement of the leptomeninges and dura mater [17,18]. More recently, DWI/FLAIR mismatch, defined as a discrepancy between the extent of diffusion restriction and FLAIR hyperintensity, has been reported as a useful diagnostic clue for this presentation [18]. In our patient, the presence of subarachnoid diffusion restriction and the DWI/FLAIR mismatch initially led to the consideration of rheumatoid meningitis. However, lobar microbleeds are not a reported finding associated with rheumatoid meningitis, and parenchymal hyperintensity on T2WI/FLAIR is observed in only approximately 30% of the cases [18,19]. Therefore, the lobar microbleeds and the infiltrative white matter lesions observed in the present case were useful in ruling out rheumatoid meningitis.

PACNS, another critical condition confined to the central nervous system that should be considered in the differential diagnosis of CAA-RI, is pathologically defined by transmural vessel wall inflammation. PACNS can affect both proximal and distal vessels and can be classified as imaging-based PACNS, which involves medium-sized vessels, and biopsy-proven PACNS (BP-PACNS), which involves small vessels [9]. The histopathologic features of BP-PACNS and ABRA are nearly indistinguishable, except for the presence of Aβ deposition that is specific to ABRA. Clinically, patients with BP-PACNS are younger at disease onset, more frequently experience headaches, and typically lack a history of intracerebral hemorrhage or cognitive impairment [9]. CSF white blood cell counts also tend to be higher in BP-PACNS than in CAA-RI [9]. Radiologic distinctions are also evident between the two entities, with subarachnoid hemorrhage, cortical superficial siderosis, and lobar microbleeds more frequently observed in patients with CAA-RI. Leptomeningeal enhancement is also more common in patients with CAA-RI, likely reflecting the inflammation related to Aβ deposition in leptomeningeal vessels, whereas nonischemic parenchymal gadolinium enhancement is more common in those with BP-PACNS, due to the inflammatory involvement of deeper white matter vessels [20]. In CAA-RI, the white matter lesions are primarily attributable to edematous changes and, therefore, lack contrast enhancement [14]. Thus, the presence of meningeal enhancement in the absence of parenchymal enhancement was useful for differentiating CAA-RI from BP-PACNS in the present case. Table 1 summarizes the differences between CAA-RI, rheumatoid meningitis, and BP-PACN.

**Table 1:** Comparison of the clinicopathologic features of CAA-RI, Rheumatoid meningitis and BP-PACNS. Abbreviations: CAA-RI, cerebral amyloid angiopathy-related inflammation; BP-PACNS, biopsy-proven primary angiitis of the central nervous system; RF, rheumatoid factor; CCP, cyclic citrullinated peptide; WMHL, white matter hyperintense lesion; LE, leptomeningeal enhancement

Parameters	CAA-RI [9]	Rheumatoid meningitis [18,19]	BP-PACNS [9]
Mean age of onset	73 years	62 years	45 years
Symptoms	Cognitive impairment is common (67%). Headache is occasionally present (31%)	Focal neurological signs (64%) and systemic symptoms (51%) are common	Cognitive impairment (75%) and headache are common (56%)
Laboratory findings	CSF pleocytosis is infrequent (25%)	CSF pleocytosis is common (85%). Either RF or anti-CCP antibodies are positive in blood	CSF pleocytosis is common (70%)
MRI features			
DWI	Acute ischemic lesions are infrequent (18%)	Subarachnoid diffusion restriction is a hallmark finding	Acute ischemic lesions are infrequent (22%)
Lobar microbleed	Lobar microbleeds are observed in almost all cases (94%)	Hemorrhagic lesions are rare	Lobar microbleeds are infrequent (26%)
WMHL on T2WI/FLAIR	WMHL extending to subcortical regions is observed in almost all cases (93%)	Parenchymal involvement is infrequent (30%)	WMHL extending to subcortical regions is common (51%). Tumefactive lesions are occasionally observed (28%)
Contrast enhancement	LE is common (70%). Nonischemic parenchymal enhancement is infrequent (16%)	LE (82%) and dural involvement (60%) are common. Parenchymal enhancement is rare	LE is infrequent (27%). Nonischemic parenchymal enhancement is common (82%)

## Conclusions

The present case illustrates a rare presentation of CAA-RI with subarachnoid diffusion restriction and dural involvement, consistent with lymphoplasmacytic infiltration. Although CAA-RI can mimic other disorders with vasculitis, such as rheumatoid meningitis and PACNS, several characteristic findings, including lobar microbleeds, infiltrative white matter lesions, and prominent leptomeningeal enhancement, supported the diagnosis of CAA-RI.

## References

[1] Kelly L, Brown C, Michalik D (2024). Clearance of interstitial fluid (ISF) and CSF (CLIC) group-part of Vascular Professional Interest Area (PIA), updates in 2022-2023. Cerebrovascular disease and the failure of elimination of amyloid-β from the brain and retina with age and Alzheimer's disease: opportunities for therapy. Alzheimers Dement.

[2] Greenberg SM, Bacskai BJ, Hernandez-Guillamon M, Pruzin J, Sperling R, van Veluw SJ (2020). Cerebral amyloid angiopathy and Alzheimer disease—one peptide, two pathways. Nat Rev Neurol.

[3] Boyle PA, Yu L, Nag S, Leurgans S, Wilson RS, Bennett DA, Schneider JA (2015). Cerebral amyloid angiopathy and cognitive outcomes in community-based older persons. Neurology.

[4] Walker L, Simpson H, Thomas AJ, Attems J (2024). Prevalence, distribution, and severity of cerebral amyloid angiopathy differ between Lewy body diseases and Alzheimer's disease. Acta Neuropathol Commun.

[5] van Veluw SJ, Benveniste H, Bakker EN (2024). Is CAA a perivascular brain clearance disease? A discussion of the evidence to date and outlook for future studies. Cell Mol Life Sci.

[6] Charidimou A, Boulouis G, Frosch MP (2022). The Boston criteria version 2.0 for cerebral amyloid angiopathy: a multicentre, retrospective, MRI-neuropathology diagnostic accuracy study. Lancet Neurol.

[7] Moussaddy A, Levy A, Strbian D, Sundararajan S, Berthelet F, Lanthier S (2015). Inflammatory cerebral amyloid angiopathy, amyloid-β-related angiitis, and primary angiitis of the central nervous system: similarities and differences. Stroke.

[8] Antolini L, DiFrancesco JC, Zedde M (2021). Spontaneous ARIA-like events in cerebral amyloid angiopathy-related inflammation: a multicenter prospective longitudinal cohort study. Neurology.

[9] Grangeon L, Boulouis G, Capron J (2024). Cerebral amyloid angiopathy-related inflammation and biopsy-positive primary angiitis of the CNS: a comparative study. Neurology.

[10] Auriel E, Charidimou A, Gurol ME (2016). Validation of clinicoradiological criteria for the diagnosis of cerebral amyloid angiopathy-related inflammation. JAMA Neurol.

[11] Theodorou A, Palaiodimou L, Malhotra K (2023). Clinical, neuroimaging, and genetic markers in cerebral amyloid angiopathy-related inflammation: a systematic review and meta-analysis. Stroke.

[12] Szalardy L, Fakan B, Maszlag-Torok R (2024). Identifying diagnostic and prognostic factors in cerebral amyloid angiopathy-related inflammation: a systematic analysis of published and seven new cases. Neuropathol Appl Neurobiol.

[13] Regenhardt RW, Thon JM, Das AS (2020). Association between immunosuppressive treatment and outcomes of cerebral amyloid angiopathy-related inflammation. JAMA Neurol.

[14] Salvarani C, Morris JM, Giannini C, Brown RD Jr, Christianson T, Hunder GG (2016). Imaging findings of cerebral amyloid angiopathy, aβ-related angiitis (ABRA), and cerebral amyloid angiopathy-related inflammation: a single-institution 25-year experience. Medicine (Baltimore).

[15] Ishida M, Shimoya N, Shibata H (2025). Amyloid β-related angiitis presenting with subarachnoid hemorrhage diagnosed by brain biopsy: a case report. NMC Case Rep J.

[16] Magaki S, Chang E, Hammond RR (2016). Two cases of rheumatoid meningitis. Neuropathology.

[17] Parsons AM, Aslam F, Grill MF, Aksamit AJ, Goodman BP (2020). Rheumatoid meningitis: clinical characteristics, diagnostic evaluation, and treatment. Neurohospitalist.

[18] Chen Zhou ZH, Hilario A, Salvador Álvarez E, Cárdenas Del Carre A, Romero Coronado J, Lechuga C, Ramos González A (2024). Radiological insights into rheumatoid meningitis - a rare central nervous system manifestation of rheumatoid arthritis: a retrospective review of six cases. Neurol Sci.

[19] Villa E, Sarquis T, de Grazia J, Núñez R, Alarcón P, Villegas R, Guevara C (2021). Rheumatoid meningitis: a systematic review and meta-analysis. Eur J Neurol.

[20] Boulouis G, de Boysson H, Zuber M (2017). Primary angiitis of the central nervous system: magnetic resonance imaging spectrum of parenchymal, meningeal, and vascular lesions at baseline. Stroke.

